# Environmental adaptation and sleep disturbance: a cross-sectional study reveals distinct metabolic risk profiles in long-term high-altitude residents versus the general population

**DOI:** 10.3389/fpsyt.2026.1789618

**Published:** 2026-07-03

**Authors:** Yi-Xiu Lin, Xiao-Yang Dong, Shu-Ming Ji, Cheng Huang, Lei Chen

**Affiliations:** 1 Department of High Altitude Medicine, Center for High Altitude Medicine, West China Hospital, Sichuan University, Chengdu, China; 2 Emergency Management Office of West China Hospital, Sichuan University, Chengdu, China; 3 Department of Clinical Research Management, West China Hospital, Sichuan University, Chengdu, China; 4 Department of Rehabilitation Medicine Center, West China Hospital, Sichuan University, Chengdu, China; 5 Department of Neurology, West China Hospital, Sichuan University, Chengdu, China

**Keywords:** environmental exposure, high-altitude, hypoxia, sleep disturbance, TyG

## Abstract

**Objective:**

Sleep disorders represent a serious global health challenge. However, the role of major environmental exposures, such as high-altitude hypoxia, and how they modify the influence of established risk factors across populations with differing susceptibility, remains poorly understood. This study aims to investigate the association between metabolic markers and sleep disorders under various environmental and adaptive conditions by comparing lowland residents with high-altitude populations (including high-altitude migrants reflecting environmental adaptation and indigenous Tibetans reflecting genetic adaptation).

**Methods:**

We analyzed data from 5,220 participants after propensity score matching. Multivariable logistic regression, restricted cubic splines, and interaction analyses were employed to examine associations between metabolic indices and sleep disturbance, adjusted for sociodemographic and lifestyle factors.

**Findings:**

Sleep disturbance prevalence differed significantly across populations (p < 0.001): lowlanders 14.9%, Plateau population 30.6% (migrants 35.0%, Tibetans 27.1%). Logistic regression revealed distinct risk patterns for sleep disturbance: plain-dwelling populations showed significant positive associations with all metabolic indices assessed (TyG index, TyG-WHtR, TyG-BMI, ABSI); The Plateau population as a whole showed significant positive associations with TyG, TyG-WHtR, and significant negative correlations with potassium; Tibetan highlanders exhibited associations only with TyG-related metabolic indicators (TyG, TyG -BMI, TyG-WHtR), with TyG exhibiting a unique nonlinear relationship; whereas plateau migrants were unrelated to other metabolic indicators except for a negative correlation with potassium. Among high-altitude migrants, depressive symptoms were associated with a stronger association between TyG-WHtR and sleep disorders; among Tibetans living at high altitudes, serum potassium was associated with a weaker association between anxiety and sleep disorders.

**Conclusion:**

Sleep disorders demonstrate heterogeneity across populations. High-altitude environments and genetic backgrounds are associated with sleep and with a modified effect of established risk factors.

**Clinical trial registration:**

https://www.chictr.org.cn/searchprojEN.html, identifier ChiCTR1900024623.

## 1 Introduction

Sleep is fundamental to human physiological and psychological function, with critical roles in cellular repair, metabolic clearance, immune regulation, emotional stability, and cognition (1–4). Inadequate sleep is consistently associated with adverse health outcomes including cardiovascular disease, cognitive impairment, and elevated all-cause mortality (1), and is recognized as a determinant of brain health by the European Academy of Neurology and a component of cardiovascular health by the American Heart Association (5, 6). Despite this recognition, sleep disorders present a growing global challenge, with insomnia affecting 10-43% of the general population (7, 8). The etiology of sleep disturbances is inherently multifactorial, involving endogenous factors (age, sex, genetic predisposition, metabolic profile) that establish basal sleep-wake architecture, and exogenous influences (psychological stress, environmental exposures, socioeconomic pressures) that dynamically modulate these foundations (9–12). While substantial evidence exists for individual risk factors, a critical knowledge gap remains in understanding how these determinants interact within specific environmental and genetic contexts to shape sleep phenotypes, as most studies examine factors in isolation, leaving their systemic interplay poorly characterized.

Insulin resistance (IR) is recognized as a key contributor to sleep disorders (10). The triglyceride glucose (TyG) index, a well-validated and easily obtainable surrogate marker of IR, correlates strongly with gold-standard measures and predicts sleep impairment and poor sleep quality across diverse populations (13–15). Obesity also elevates sleep disturbance risk, with meta-analyses reporting a 33% increased risk among overweight individuals (BMI ≥ 25) (16), potentially mediated by structural, metabolic, inflammatory, and autonomic pathways (17). To capture metabolic dysregulation more comprehensively, integrated indices such as TyG-BMI and TyG-waist-to-height ratio (TyG-WHtR) have been developed, reflecting dyslipidemia, glycemia and adiposity patterns. Although these show prognostic value in cardiometabolic research (18, 19), their association with sleep outcomes remains inadequately explored, underscoring the need for further investigation.

Environmental exposure is another well-established modulator of sleep. The external environment exerts a critical influence on sleep through a complex interplay of physical and chemical factors. Multifaceted environmental stressors—ranging from light-at-night and noise pollution to extreme temperatures, airborne pollutants, and the hypoxic conditions of high altitudes—can collectively disrupt circadian rhythms and impair sleep continuity and depth (11, 20, 21). Increasing economic development has led to a growing number of individuals converging on high-altitude regions for work and tourism, with over 10 million people now residing there long-term (22). High-altitude environments are characterized by a constellation of distinctive natural conditions including hypoxia, reduced atmospheric pressure, marked diurnal temperature variations, intense solar radiation, and aridity, all of which exert profound physiological effects (10). As the organ most sensitive to oxygen deprivation, the brain demonstrates particular vulnerability to functional impairment under high-altitude conditions (23). While the hypoxic and hypobaric environment at high altitude is a well-established risk factor for sleep disturbances, the precise magnitude of its impact on human sleep health remains inadequately quantified, presenting a significant public health concern (24). Empirical evidence indicates that sleep quality, architecture, and continuity substantially deteriorate following high-altitude exposure. Newcomers to high elevations frequently report fatigue, increased nightmare frequency, sleep fragmentation, and morning dizziness. These symptoms collectively exacerbate daytime somnolence and fatigue (25), intensify manifestations of acute mountain sickness, and impair both short-term memory and work capacity (23, 26), thereby contributing to broader cognitive impairment (27). Furthermore, chronic sleep apnea at altitude shows established associations with chronic mountain sickness and elevated cardiovascular disease incidence (28).

The elevated prevalence of sleep disorders in high-altitude populations compared to plain-dwelling groups has attracted considerable attention. A comparative study in China revealed a chronic insomnia rate of 41.54% among elderly residents at 2,300 meters, significantly higher than the 18.76% observed at low-altitude sites (29). Kan et al. demonstrated a significant increase in Pittsburgh Sleep Quality Index (PSQI) scores among migrants at 4,500 meters, with emerging symptoms including difficulty falling asleep, frequent nighttime awakenings, and snoring (30). In a separate investigation involving 170 participants, high-altitude residents showed a substantially higher prevalence of sleep apnea compared to lowland controls (77% vs. 54%, p < 0.001), with the high-altitude group exhibiting further reduced oxygen saturation (SPO_2_) levels during sleep (31). Sleep quality further declines with increasing elevation (32). In addition, the Qinghai-Tibet Plateau has a human habitation history of 25,000 years, and the long-term plateau environment has posed a systemic physiological challenge (33). The Tibetan population has evolved unique genetic adaptations to hypoxia. The strong selection of genes such as EPAS1 and EGLN1 has resulted in only a slight increase in, or maintenance of, hemoglobin concentration within the normal range observed in plains, even in high-altitude environments (34). This is compensated for by an increased respiratory rate and lung capacity, which reduces the incidence of chronic mountain sickness (CMS) (35). Whether these adaptive changes to hypoxia influence sleep disorders in high-altitude Tibetans remains to be explored. Consistent with the role of genetic adaptation, current research finds that lowland immigrants to high altitude generally exhibit poorer sleep outcomes than native highlanders (36), underscoring the potential role of long-term genetic adaptation in modulating environmental effects on sleep.

IR and high-altitude exposure are both important determinants of sleep, but most studies have examined them separately and rarely combined them. The impact of high-altitude environments varies across populations: migrants rely on short-term acclimatization, whereas Tibetans have long-term genetic adaptation. These differences may alter the relationship between metabolic indicators and sleep disorders. Therefore, focusing only on the direct effects of metabolic factors or environmental exposure may overlook the important dimension of metabolism–environment interaction.

This study systematically examined sleep quality in lowlanders and high-altitude populations. We first compared lowlanders with the combined high-altitude population (migrants plus Tibetans), then disaggregated the high-altitude group into migrants and Tibetans to investigate how metabolic indices associate with sleep disturbance under different adaptation modes (acclimatization vs. genetic adaptation).

## 2 Methods

### 2.1 Study design and data source

This cross-sectional analysis was conducted using data from the 2021 survey wave of the Western China Natural Population Cohort Study (WCNPCS). The WCNPCS is a large-scale, community-based prospective cohort established in 2019 to investigate health determinants in the adult population of Western China. Data were obtained through a standardized protocol that included face-to-face interviews, structured questionnaires, and physical examinations (37).

### 2.2 Ethics statement

Ethical approval for this study was obtained from the Ethics Committee of West China Hospital, Sichuan University [ethical approval number: 2020 Review (145)] and the trial was registered with the Chinese Clinical Trial Registry (ChiCTR1900024623). The study was conducted in full accordance with the ethical principles of the Declaration of Helsinki and relevant local regulations. Written informed consent was secured from all participants prior to their enrollment.

### 2.3 Study population and propensity score matching

**Figure 1:**
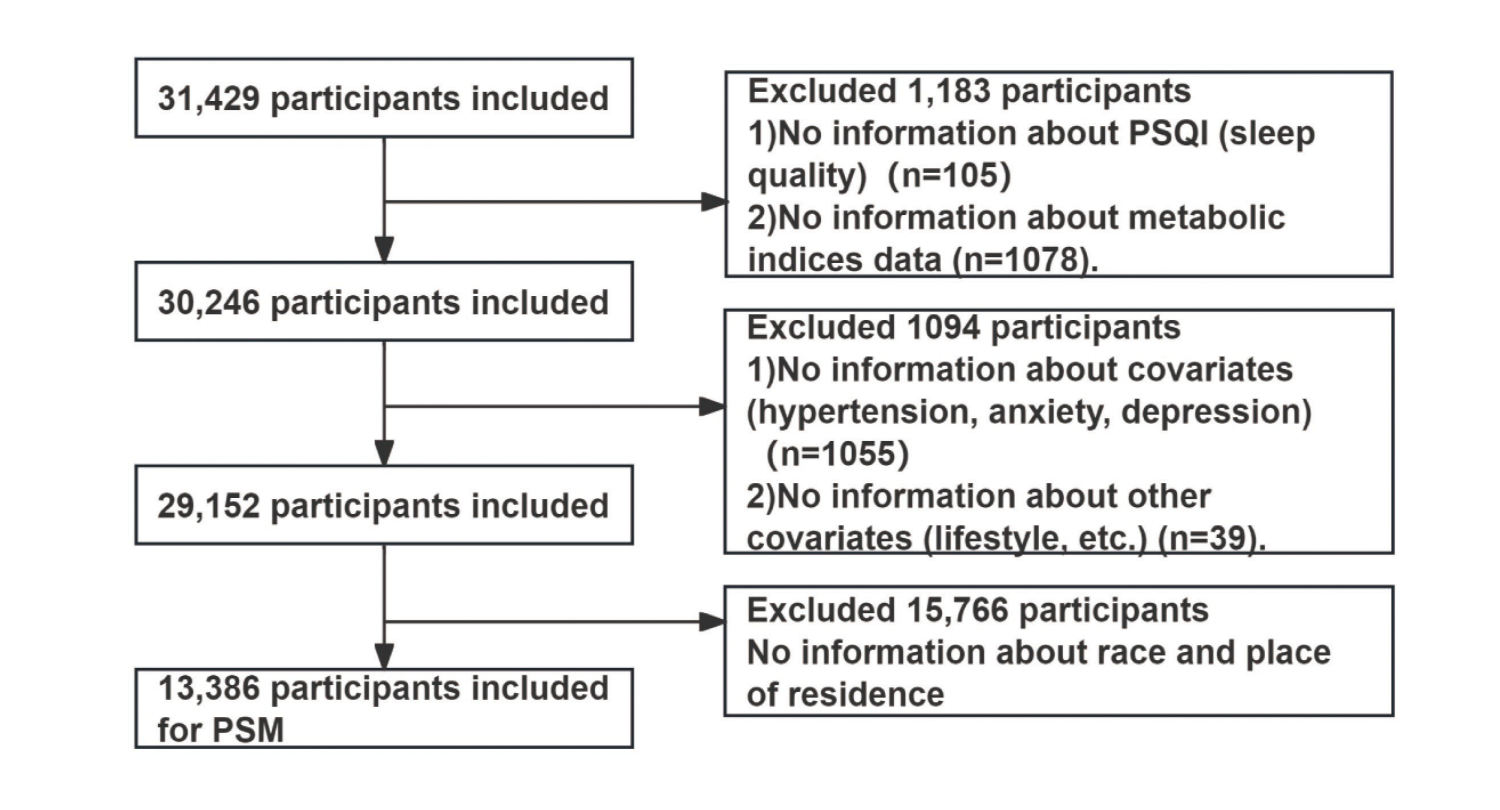
Study population selection flowchart. Flow diagram illustrating the participant inclusion and exclusion process for the three study groups: lowlanders, high-altitude migrants, and Tibetan highlanders.

The WCNPCS cohort recruited permanent residents of the participating communities through sequential cluster sampling. From the overall WCNPCS 2021 survey wave, we initially identified 31,429 potential participants. After applying the inclusion criteria (adults with complete data on ethnicity and all study variables), 13,386 individuals were included in the primary analytical sample (**Figure 1**). For the purpose of this analysis, participants were categorized into three groups based on ethnicity and altitude of residence: Lowlanders (low-altitude residents), High-altitude Migrants (people who migrated to high altitude), and Tibetan Highlanders (Tibetans with long-term ancestral residence at high altitude). This categorization was fundamental to addressing our research question, as the primary sample distinction lay between the lowlanders and the combined high-altitude groups (migrants and indigenous Tibetan highlanders).

Altitude was defined as >2500 m for high altitude and <1500 m for low altitude. Permanent residency was ensured because the WCNPCS cohort only enrolls permanent residents. The following three groups were defined for analysis: Lowlanders: Chinese participants who had resided at <1500 m for ≥10 years; Tibetan highlanders: Participants with Tibetan ethnicity on both identity card and hukou, residing as permanent residents at >2500 m. In these regions, Tibetans are considered indigenous with long-term ancestral residence (for thousands of years), representing genetic adaptation (38); High-altitude migrants: All other high-altitude permanent residents (non-Tibetan). Because non-Tibetans in these areas are typically not indigenous but have moved there for work or other reasons.

To ensure comparability and mitigate confounding due to this substantial sample size imbalance, we performed propensity score matching (PSM) (39). Participants from the high-altitude region (the exposure group of interest) were matched with participants from the plains (the control group). The propensity score was estimated using a logistic regression model that included the following pre-specified covariates: age, sex, education level, marital status, smoking status, alcohol consumption, hypertension.

We employed a 1:2 nearest-neighbor greedy matching algorithm without replacement, applying a caliper width of 0.2 times the standard deviation of the propensity score logit. This procedure successfully matched 1,764 treated participants (high-altitude residents) with 3,456 controls (lowlanders), achieving a control-to-treated ratio of approximately 1.96:1. This slight deviation from the theoretical 2:1 target reflects the necessary trade-off to prioritize covariate balance over sample size by excluding low-quality matches. The final balanced analytical sample comprised 5,220 participants, categorized into two distinct groups: lowlanders (n=3,456) and plateau population (n=1764) (**Table 1**).

**Table 1:** Baseline characteristics of the participants after propensity score matching (demographic and lifestyle factors).

Variable	Before propensity score matching				After propensity score matching			
	Lowlanders	Plateau population	P values	SMD	Lowlanders	Plateau population	P values	SMD
Sample size	10511	2875			3456	1764		
Age, mean ± SD	57.58 ± 11.88	58.03 ± 12.77	0.084	0.037	59.36 ± 11.23	58.96 ± 12.30	0.259	0.033
Sex, n (%)			0.038	0.021			0.184	0.019
Female	6720 (63.9%)	1777 (61.8%)			2372 (68.6%)	1178 (66.8%)		
Male	3791 (36.1%)	1098 (38.2%)			1084 (31.4%)	586 (33.2%)		
Marital status, n (%)			<0.001	0.086			0.019	0.026
Married	9449 (89.9%)	2336 (81.3%)			2922 (84.5%)	1446 (82.0%)		
Unmarried	1062 (10.1%)	539 (18.7%)			534 (15.5%)	318 (18.0%)		
Smoking status, n (%)			<0.001	0.071			0.297	0.013
No	7858 (74.8%)	2353 (81.8%)			2773 (80.2%)	1393 (79.0%)		
Yes	2653 (25.2%)	522 (18.2%)			683 (19.8%)	371 (21.0%)		
Drinking status, n (%)			<0.001	0.071			0.045	0.027
No	7035 (66.9%)	2129 (74.1%)			2535 (73.4%)	1247 (70.7%)		
Yes	3476 (33.1%)	746 (25.9%)			921 (26.6%)	517 (29.3%)		
BMI category, n (%)			<0.001	0.064			0.167	0.020
Normal weight	4946 (47.1%)	1205 (41.9%)			1473 (42.6%)	751 (42.6%)		
Overweight	4118 (39.2%)	1089 (37.9%)			1377 (39.8%)	669 (37.9%)		
Obese	1447 (13.8%)	581 (20.2%)			606 (17.5%)	344 (19.5%)		

Matching variables: age, sex, education (edu), marital status (mar), smoking (smok), drinking (drink), BMI and hypertension. Data are presented as mean ± standard deviation for continuous variables and n (%) for categorical variables. P-values were derived from ANOVA or chi-square tests. SMD, standardized mean difference. Matching variables included age, sex, education, marital status, smoking, drinking, BMI, and hypertension.

### 2.4 Variable definitions

#### 2.4.1 Primary variables

The primary outcome was sleep disturbance, operationalized as a global score >7 on the Pittsburgh Sleep Quality Index (PSQI), a validated 7-component questionnaire assessing sleep quality over the preceding month (40). The PSQI cutoff of 7, commonly used in epidemiology, has been validated in Chinese populations, showing 98.3% sensitivity and 90.2% specificity for sleep disturbance (41–43). The Cronbach’s alpha coefficient was 0.74, indicating acceptable internal consistency for group-level comparisons. To investigate the metabolic-sleep relationship, we selected a panel of validated metabolic indices as key independent variables. Each index was calculated as follows:

Triglyceride-glucose (TyG) index (44): A validated surrogate marker of insulin resistance, computed using the formula:
TyG = ln [fasting triglycerides (mg/dL) × fasting glucose (mg/dL)/2]
TyG-Body Mass Index (TyG-BMI): An integrated metric combining insulin resistance and overall adiposity, derived as:
TyG-BMI = TyG index × BMI (kg/m²)
TyG-Waist-to-Height Ratio (TyG-WHtR): A composite measure integrating insulin resistance and central adiposity, calculated as:
TyG-WHtR = TyG index × (waist circumference [cm]/height [cm])
A Body Shape Index (ABSI): A standardized metric of abdominal obesity adjusted for height and weight, calculated as:
ABSI = waist circumference (m)/[height (m) ^ (2/3) × weight (kg) ^ (1/2)]

Serum Potassium (K^+^): Measured via venous blood, reflecting electrolyte balance and potential links to metabolic and cardiovascular pathways.

#### 2.4.2 Covariates

Covariates included sociodemographic, behavioral, and health-related factors associated with the primary outcomes in previous literature.

Sociodemographic factors included sex (male/female), age (continuous, in years), education level (categorized as: no formal education/primary school, junior middle school, technical secondary school/high school, or junior college/bachelor’s degree or higher), and marital status (married vs. single/divorced/widowed).

Health behaviors considered were smoking status (ever smoked: yes/no) and drinking status (any lifetime use: yes/no).

Health status indicators included body mass index (<24 kg/m², 24–27.9 kg/m² and ≥28 kg/m², using the Chinese criterion for obesity) and hypertension (elevated: systolic ≥140 mmHg or diastolic ≥90 mmHg; normal: otherwise).

Psychological traits and symptoms were assessed using standardized scales: the Life Orientation Test (LOT) for optimism, the Satisfaction with Life Scale (SWLS), the Loneliness Scale (LONLY), the Generalized Anxiety Disorder-7 (GAD-7) for anxiety symptoms, and the Patient Health Questionnaire-9 (PHQ-9) for depressive symptoms (45, 46).

### 2.5 Statistical analysis

All analyses were performed using R software (version 4.5.2) and Free Statistics software V2.2.0 (47, 48), with a two-sided p-value < 0.05 considered statistically significant. After propensity score matching (2 groups: lowlanders vs. combined high-altitude population), baseline characteristics between the two matched groups were compared using independent t-tests for continuous variables and chi-square tests for categorical variables. Standardized mean differences (SMDs) were calculated to assess balance, with SMD < 0.1 considered indicative of adequate balance.

The prevalence of sleep disturbance in the matched lowlanders and combined Plateau population was compared using the chi-square test. For comparison of sleep disturbance prevalence among the three subgroups (lowlanders, high-altitude migrants, and Tibetan highlanders), the chi-square test was used, followed by pairwise *post-hoc* comparisons with Bonferroni correction (α’ = 0.0167) when the overall test was significant.

The assumed causal relationships among key variables are illustrated in a directed acyclic graph (DAG, **Supplementary Figure 1**). In this framework, psychological symptoms lie on the pathway from metabolic indices to sleep disturbance. Adjusting for them in Model 3 allows estimation of the association between metabolic indices and sleep disturbance independent of psychological symptoms, while interaction tests (e.g., X × M) assess effect modification without introducing bias under this causal structure.

We employed multivariable logistic regression to assess the associations between continuous metabolic indices (TyG, TyG-BMI, TyG-WHtR, ABSI, and serum potassium) and sleep disturbance (defined as PSQI > 7). To transparently delineate the contribution of different confounder domains, we constructed three sequential models for each population subgroup: Model 1 (Crude) estimated the unadjusted association; Model 2 adjusted for core sociodemographic (age, sex), lifestyle (smoking, alcohol, physical exercise), and stable psychological traits (optimism/LOT, life satisfaction/SWLS, loneliness/LONLY), thereby testing the association after accounting for background disposition and behavior; and Model 3 (Fully Adjusted) further controlled for dynamic clinical states—including hypertension, and current anxiety (GAD-7) and depressive (PHQ-9) symptoms—to address whether metabolic indices provide information on sleep disturbance beyond the profound influence of co-occurring psychological symptoms.

To move beyond simple linear assumptions and uncover group-specific nuances, we conducted two targeted in-depth analyses. First, we used restricted cubic splines (RCS) with 4 knots (placed at the 10th, 33rd, 67th, and 90th percentiles) to flexibly model the continuous relationship between each metabolic index and the log-odds of sleep disturbance. The median value (50th percentile) of each metabolic index within each subgroup was used as the reference (OR = 1) (49). This was essential for testing whether threshold effects or nonlinear dynamics—potentially reflecting underlying physiological saturation points or compensatory mechanisms—differed across populations, particularly in the unique high-altitude groups. Second, we pre-specified and tested interaction terms to evaluate whether the strength of the metabolic-sleep association was modified by key physiological and psychological states, directly probing our hypothesis that this link is not uniform but contingent on an individual’s clinical profile. Specifically, we examined whether hypertension (a state of heightened cardiovascular stress) amplified the risk associated with metabolic indices, and whether co-existing depressive (PHQ-9) or anxiety (GAD-7) symptoms acted as potent effect modifiers, positing that the combined burden of metabolic dysregulation and psychological distress may be synergistic. To assess the discriminative ability of each metabolic index regarding sleep disturbance and contextualize the observed modest odds ratios, we calculated the area under the curve (AUC) of the receiver operating characteristic for the fully adjusted model (**Supplementary Table 1**).

Finally, to ensure the robustness of our primary findings, we conducted sensitivity analyses by varying the caliper widths in propensity score matching. In addition to the primary caliper of 0.2 used in our main analysis, we tested alternative calipers of 0.15, 0.25, and 0.3.

A signed Statistical Review Declaration from Prof. Deying Kang (Center of Biostatistics, West China Hospital) is available as **Supplementary Document 1**.

## 3 Results

### 3.1 Study population characteristics after matching

After propensity score matching, the analysis included 3,456 lowlanders and 1,764 high-altitude residents (comprising 769 high-altitude migrants and 995 Tibetan highlanders). Matching variables comprised age, sex, education level, marital status, smoking status, drinking status, BMI category, and hypertension status. Post-matching, standardized mean differences (SMDs) for all covariates were below 0.2, and most P-values exceeded 0.05, indicating adequate balance across groups and effective reduction of selection bias (**Tables 1**, **2**; **Supplementary Figure 2**).

**Table 2:** Baseline characteristics of the participants after propensity score matching (clinical, metabolic, and psychological measures).

Variable	Before propensity score matching				After propensity score matching			
	Lowlanders	Plateau population	P values	SMD	Lowlanders	Plateau population	P values	SMD
Hypertension status, n (%)			<0.001	0.054			0.182	0.02
Hypertension	4212 (40.1%)	1308 (45.5%)			1493 (43.2%)	797 (45.2%)		
Non-hypertension	6299 (59.9%)	1567 (54.5%)			1963 (56.8%)	967 (54.8%)		
LOT score, mean ± SD	20.40 ± 3.13	19.95 ± 3.66	<0.001	0.133	20.32 ± 3.19	20.08 ± 3.67	0.018	0.071
SWLS score, mean ± SD	24.18 ± 4.65	24.48 ± 4.85	0.004	0.062	24.10 ± 4.78	24.74 ± 4.80	<0.001	0.136
Loneliness score, mean ± SD	3.20 ± 0.77	3.33 ± 0.90	<0.001	0.148	3.24 ± 0.82	3.28 ± 0.84	0.102	0.048
Anxiety score, mean ± SD	0.81 ± 2.26	0.86 ± 2.53	0.300	0.022	0.89 ± 2.44	1.33 ± 3.03	<0.001	0.159
Depression score, mean ± SD	0.87 ± 2.14	1.35 ± 3.08	<0.001	0.178	0.98 ± 2.25	1.44 ± 3.17	<0.001	0.167
Serum potassium (K^+^), mean ± SD	4.08 ± 0.38	4.09 ± 0.28	0.019	0.045	4.12 ± 0.41	4.16 ± 0.37	<0.001	0.102
TyG index, mean ± SD	2.02 ± 0.63	1.89 ± 0.64	<0.001	0.208	2.03 ± 0.64	1.98 ± 0.59	0.004	0.084
TyG-BMI index, mean ± SD	50.37 ± 32.13	47.94 ± 19.57	<0.001	0.091	51.23 ± 19.80	50.05 ± 18.31	0.033	0.062
TyG-WHtR, mean ± SD	1.02 ± 0.37	1.00 ± 0.38	0.016	0.051	1.04 ± 0.39	1.05 ± 0.36	0.494	0.02
A Body Shape Index, mean ± SD	10.12 ± 0.98	10.53 ± 0.91	<0.001	0.43	10.17 ± 0.78	10.52 ± 0.94	<0.001	0.397
Sleep disturbance, n (%)			<0.001	0.081			<0.001	0.157
Normal sleep	9012 (85.7%)	2232 (77.6%)			2942 (85.1%)	1225 (69.4%)		
Sleep disturbance	1499 (14.3%)	643 (22.4%)			514 (14.9%)	539 (30.6%)		

Matching variables: age, sex, education (edu), marital status (mar), smoking (smok), drinking (drink), BMI and hypertension. Data are presented as mean ± standard deviation for continuous variables and n (%) for categorical variables. P-values were derived from ANOVA or chi-square tests. SMD, standardized mean difference. Matching variables included age, sex, education, marital status, smoking, drinking, BMI, and hypertension.

### 3.2 Prevalence of sleep disturbance

**Figure 2:**
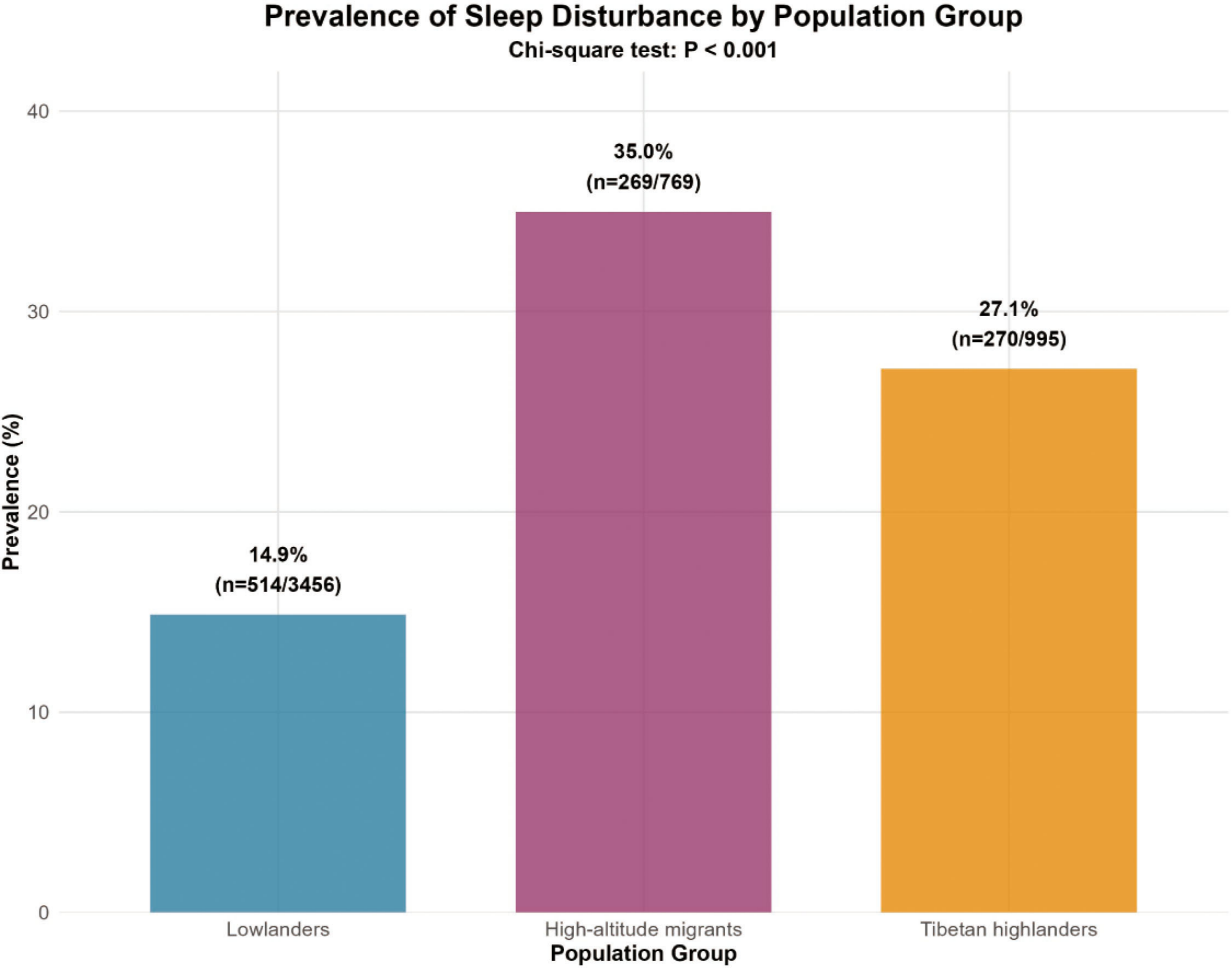
Prevalence of sleep disturbance across the three study populations. Bar graph showing the proportion of participants with sleep disturbance in each study group. Error bars represent 95% confidence intervals.

The prevalence of sleep disturbance was 14.9% in lowlanders, 30.6% in the combined Plateau population (p < 0.001, **Table 2**). When further disaggregated, the prevalence was 35.0% in high-altitude migrants, and 27.1% in Tibetan highlanders (**Figure 2**). The chi-square test showed a significant overall difference (χ² = 194.96, df = 2, *p* < 0.001). Bonferroni-corrected pairwise comparisons confirmed significant differences between all three groups (all *p* ≤ 0.0014).

### 3.3 Associations of metabolic indices with sleep disturbance

The associations between metabolic indices and sleep disturbance exhibited distinct patterns across the four populations in the multivariable logistic regression analysis (**Table 3**).

**Table 3:** Logistic regression analysis of metabolic indices and sleep disturbance across population subgroups (main effects model).

Independent variable	Model	OR	95% CI	p.value	OR	95% CI	p.value	OR	95% CI	p.value	OR	95% CI	p.value
		Lowlanders			Plateau population			High-altitude migrants			Tibetan Plateau population		
TyG	model 1	1.13	(1.03,1.23)	**0.006**	1.166	(1.05,1.29)	**0.004**	1.09	(0.93,1.28)	0.277	1.18	(1.01,1.36)	**0.033**
TyG-BMI Index		1.11	(1.02,1.21)	**0.018**	1.092	(0.98,1.21)	0.102	1.02	(0.87,1.19)	0.845	1.13	(0.98,1.30)	0.103
TyG-WHtR		1.16	(1.06,1.27)	**0.001**	1.144	(1.03,1.27)	**0.012**	1.08	(0.92,1.26)	0.344	1.16	(1.00,1.34)	0.053
A Body Shape Index		1.35	(1.22,1.50)	**0**	1.08	(0.97,1.2)	0.143	1.11	(0.98,1.28)	0.125	1.03	(0.90,1.18)	0.682
Serum Potassium		0.98	(0.9,1.08)	0.713	0.858	(0.77,0.96)	**0.008**	0.78	(0.67,0.91)	**0.002**	0.96	(0.82,1.12)	0.624
TyG	model 2	1.11	(1.01,1.22)	**0.027**	1.178	(1.06,1.31)	**0.003**	1.07	(0.91,1.27)	0.398	1.23	(1.05,1.43)	**0.01**
TyG-BMI Index		1.09	(0.99,1.20)	0.064	1.096	(0.98,1.22)	0.102	0.99	(0.84,1.17)	0.89	1.16	(1.00,1.35)	0.053
TyG-WHtR		1.12	(1.02,1.23)	**0.016**	1.139	(1.02,1.27)	**0.018**	1.04	(0.88,1.22)	0.665	1.19	(1.02,1.39)	**0.028**
A Body Shape Index		1.23	(1.10,1.38)	**0**	1.042	(0.95,1.15)	0.406	1.05	(0.92,1.21)	0.456	1.01	(0.87,1.16)	0.93
Serum Potassium		1.01	(0.92,1.11)	0.811	0.865	(0.77,0.97)	**0.014**	0.78	(0.67,0.92)	**0.004**	0.98	(0.83,1.14)	0.761
TyG	model 3	1.13	(1.03,1.25)	**0.012**	1.19	(1.06,1.33)	**0.003**	1.09	(0.92,1.30)	0.32	1.24	(1.05,1.46)	**0.01**
TyG-BMI Index		1.12	(1.01,1.23)	**0.025**	1.112	(0.99,1.25)	0.076	1	(0.84,1.20)	0.963	1.19	(1.01,1.40)	**0.033**
TyG-WHtR		1.15	(1.04,1.27)	**0.006**	1.153	(1.03,1.3)	**0.017**	1.06	(0.89,1.25)	0.541	1.21	(1.03,1.42)	**0.022**
A Body Shape Index		1.26	(1.12,1.42)	**0**	1.035	(0.93,1.15)	0.530	1.06	(0.92,1.22)	0.453	1	(0.86,1.16)	0.995
Serum Potassium		1	(0.91,1.1)	0.995	0.858	(0.76,0.96)	**0.010**	0.77	(0.65,0.92)	**0.003**	0.96	(0.81,1.14)	0.668

Model 1, no covariates were adjusted.

Model 2, adjusted for Age, Sex, Marital status, and Education, smoking status, alcohol consumption, BMI, scores on the Life Orientation Test (LOT), the Satisfaction with Life Scale (SWLS), and the Loneliness Scale (LONLY).

Model 3, adjusted for Age, Sex, Marital status, and Education, smoking status, alcohol consumption, scores on the Life Orientation Test (LOT), the Satisfaction with Life Scale (SWLS), and the Loneliness Scale (LONLY), hypertension, anxiety symptoms (assessed by the GAD-7), and depressive symptoms (assessed by the PHQ-9).

OR, odds ratio; CI, confidence interval. Model 1, unadjusted. Model 2, adjusted for age, sex, marital status, education, smoking, alcohol consumption, BMI, LOT, SWLS, and loneliness. Model 3, further adjusted for hypertension, anxiety, and depressive symptoms. Bold indicates P < 0.05.

In the Lowlanders, multiple metabolic indices showed consistent and statistically significant associations with higher odds of sleep disturbance across all three models. For instance, in the fully adjusted Model 3, each unit increase in the TyG index (OR: 1.13, 95% CI: 1.03-1.25), TyG-BMI index (OR: 1.12, 95% CI: 1.01-1.23), TyG-WHtR index (OR: 1.15, 95% CI: 1.04-1.27), and A Body Shape Index (ABSI) (OR: 1.26, 95% CI: 1.12-1.42) was associated with an increased risk of sleep disturbance.

In the combined Plateau population (high-altitude migrants and Tibetan highlanders analyzed together), the TyG index (OR: 1.19, 95% CI: 1.06-1.33), TyG-WHtR (OR: 1.15, 95% CI: 1.03-1.30), and serum potassium (OR: 0.86, 95% CI: 0.76-0.96) showed significant associations with sleep disturbance in Model 3, while TyG-BMI and ABSI did not reach statistical significance.

When the two high-altitude groups were analyzed separately, marked heterogeneity emerged. In high-altitude migrants, none of the metabolic indices (TyG, TyG-BMI, TyG-WHtR, or ABSI) were significantly associated with sleep disturbance in any adjusted model (Model 2 or 3). Notably, serum potassium demonstrated a protective effect, with each unit increase associated with approximately 23% lower odds of sleep disturbance in Model 3 (OR: 0.77, 95% CI: 0.65-0.92).

In Tibetan highlanders, a different pattern was observed. The TyG index (OR: 1.24, 95% CI: 1.05-1.46), TyG-BMI index (OR: 1.19, 95% CI: 1.01-1.40), and TyG-WHtR index (OR: 1.21, 95% CI: 1.03-1.42) were all significantly associated with sleep disturbance in Model 3. However, ABSI showed no significant association (OR: 1.00, 95% CI: 0.86-1.16), and serum potassium was not significant (OR: 0.96, 95% CI: 0.81-1.14).

### 3.4 Moderating effects of psychological and physiological factors

The analysis of moderating effects revealed that the associations between metabolic indices and sleep disturbance were significantly modified by psychological and physiological factors, with notable differences across the three groups (**Table 4**). Specifically, hypertension significantly moderated the relationships between several metabolic indices (TyG and TyG-WHtR) and sleep disturbance in both the lowlanders and Tibetan highlanders, but not in the high-altitude migrants group. In the high-altitude migrants, a significant interaction was observed between the TyG-WHtR index and depressive symptoms (interaction OR: 1.94, 95% CI: 1.08–3.71), indicating that the adverse effect of this metabolic index on sleep disturbance was stronger among individuals with more severe depression. Conversely, in the Tibetan highlanders, a significant interaction emerged between serum potassium levels and anxiety symptoms (interaction OR: 0.59, 95% CI: 0.36–0.97), suggesting a protective effect of higher potassium that varied by anxiety severity.

**Table 4:** Analysis of the moderating effects of psychological and physiological factors on sleep disturbance across population subgroups.

Interaction term	Independent variable	OR	95%CI	p.value	OR	95%CI	p.value	OR	95%CI	p.value
		Lowlanders			High-altitude migrants			Tibetan highlanders		
Depression	TyG	1.11	(1.00,1.22)	0.057	1.02	(0.85,1.22)	0.862	1.23	(1.04,1.46)	**0.015**
	Depression	8.57	(6.17,11.97)	**0.000**	3.83	(2.19,6.83)	**0.000**	5.82	(3.47,10.02)	**0.000**
	TyG * Depression	1.08	(0.81,1.45)	0.596	1.62	(0.91,3.02)	0.114	1.11	(0.64,1.97)	0.728
	TyG-BMI Index	1.10	(0.99,1.22)	0.067	0.93	(0.77,1.11)	0.417	1.19	(1.01,1.40)	**0.041**
	Depression	8.59	(6.19,11.98)	**0.000**	4.09	(2.34,7.31)	**0.000**	5.95	(3.54,10.31)	**0.000**
	TyG-BMI Index * Depression	0.98	(0.75,1.29)	0.893	1.78	(1.00,3.36)	0.060	1.12	(0.66,1.97)	0.692
	TyG-WHtR	1.12	(1.01,1.24)	**0.034**	0.97	(0.81,1.15)	0.698	1.21	(1.02,1.42)	**0.026**
	Depression	8.59	(6.18,11.99)	**0.000**	3.80	(2.16,6.80)	**0.000**	5.75	(3.47,9.77)	**0.000**
	TyG-WHtR * Depression	1.05	(0.80,1.39)	0.729	1.94	(1.08,3.71)	**0.034**	1.06	(0.61,1.89)	0.832
	A Body Shape Index	1.21	(1.07,1.37)	**0.003**	1.01	(0.88,1.16)	0.939	1.00	(0.86,1.17)	0.983
	Depression	8.91	(6.38,12.54)	**0.000**	3.52	(1.98,6.34)	**0.000**	5.54	(3.39,9.17)	**0.000**
	A Body Shape Index * Depression	1.25	(0.91,1.75)	0.184	1.62	(0.96,2.91)	0.088	0.99	(0.65,1.50)	0.948
	Serum Potassium	1.00	(0.90,1.11)	0.994	0.78	(0.65,0.93)	**0.006**	0.97	(0.81,1.16)	0.717
	Depression	8.58	(6.17,11.97)	**0.000**	4.18	(2.41,7.46)	**0.000**	5.54	(3.42,9.12)	**0.000**
	Serum Potassium * Depression	1.06	(0.78,1.44)	0.698	0.98	(0.59,1.61)	0.948	0.97	(0.60,1.57)	0.896
Anxiety	TyG	1.11	(1.00,1.23)	**0.039**	1.12	(0.93,1.34)	0.238	1.24	(1.05,1.46)	**0.011**
	Anxiety	3.15	(2.31,4.28)	**0.000**	2.72	(1.69,4.42)	**0.000**	3.24	(1.95,5.45)	**0.000**
	TyG * Anxiety	0.97	(0.73,1.27)	0.830	0.81	(0.51,1.27)	0.351	0.96	(0.57,1.66)	0.889
	TyG-BMI Index	1.10	(0.99,1.22)	0.063	1.02	(0.85,1.23)	0.811	1.19	(1.01,1.39)	**0.038**
	Anxiety	3.16	(2.32,4.29)	**0.000**	2.67	(1.66,4.32)	**0.000**	3.22	(1.94,5.40)	**0.000**
	TyG-BMI Index * Anxiety	0.94	(0.71,1.23)	0.671	0.80	(0.51,1.27)	0.341	0.93	(0.54,1.60)	0.783
	TyG-WHtR	1.12	(1.02,1.24)	**0.023**	1.06	(0.89,1.27)	0.494	1.23	(1.04,1.44)	**0.014**
	Anxiety	3.15	(2.31,4.28)	**0.000**	2.70	(1.68,4.40)	**0.000**	3.14	(1.9,5.24)	**0.000**
	TyG-WHtR * Anxiety	0.96	(0.73,1.26)	0.784	0.85	(0.54,1.34)	0.472	0.83	(0.48,1.43)	0.493
	A Body Shape Index	1.23	(1.09,1.39)	**0.001**	1.02	(0.89,1.18)	0.799	1.07	(0.92,1.24)	0.394
	Anxiety	3.16	(2.31,4.29)	**0.000**	2.36	(1.43,3.90)	**0.001**	3.38	(2.07,5.56)	**0.000**
	A Body Shape Index * Anxiety	0.99	(0.72,1.37)	0.934	1.43	(0.88,2.42)	0.165	0.62	(0.36,1.02)	0.066
	Serum Potassium	1.00	(0.9,1.10)	0.930	0.77	(0.64,0.92)	**0.004**	1.04	(0.87,1.24)	0.653
	Anxiety	3.15	(2.31,4.28)	**0.000**	2.66	(1.65,4.31)	**0.000**	3.34	(2.04,5.49)	**0.000**
	Serum Potassium * Anxiety	1.09	(0.81,1.46)	0.580	1.19	(0.75,1.86)	0.451	0.59	(0.36,0.97)	**0.040**

Interaction terms were included to test whether depression or anxiety moderates the association between metabolic indices and sleep disturbance. Models were adjusted for age, sex, marital status, education, smoking, alcohol consumption, BMI, LOT, SWLS, loneliness, hypertension, and the respective psychological factor (depression or anxiety). Bold indicates P < 0.05. *Indicates interaction term between two variables.

#### 3.4.1 Nonlinear associations and dose-response relationships

**Figure 3:**
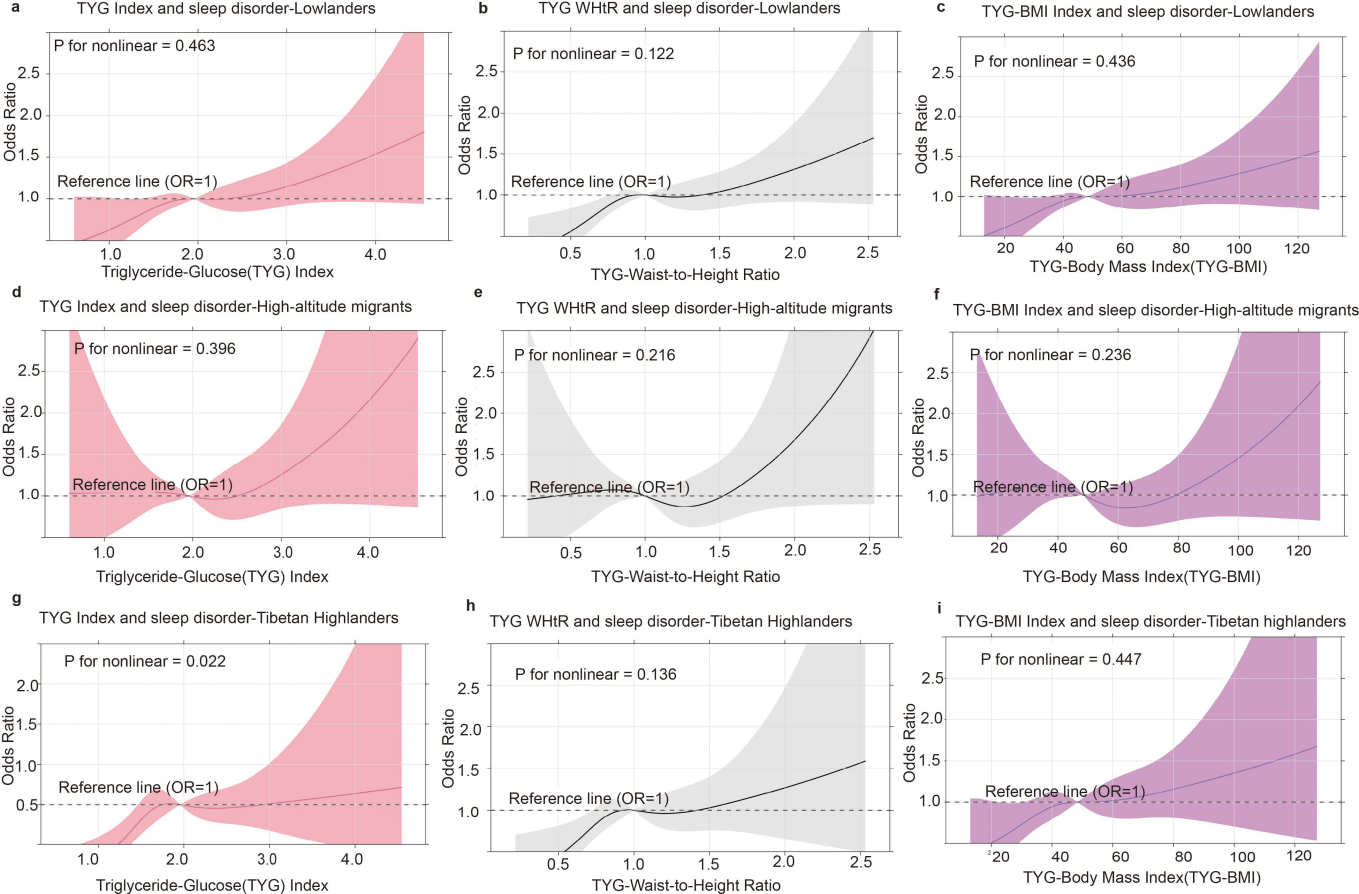
Restricted cubic spline plots depict the dose-response relationships between metabolic indices and sleep disturbance risk across three population groups. **(a)** TyG index and sleep disturbance risk in lowlanders; **(b)** TyG-WHtR and sleep disturbance risk in lowlanders; **(c)** TyG-BMI and sleep disturbance risk in lowlanders; **(d)** TyG index and sleep disturbance risk in high-altitude migrants; **(e)** TyG-WHtR and sleep disturbance risk in high-altitude migrants; **(f)** TyG-BMI and sleep disturbance risk in high-altitude migrants; **(g)** TyG index and sleep disturbance risk in Tibetan highlanders; **(h)** TyG-WHtR and sleep disturbance risk in Tibetan highlanders; **(i)** TyG-WHtR and sleep disturbance risk in Tibetan highlanders.

**Figure 4:**
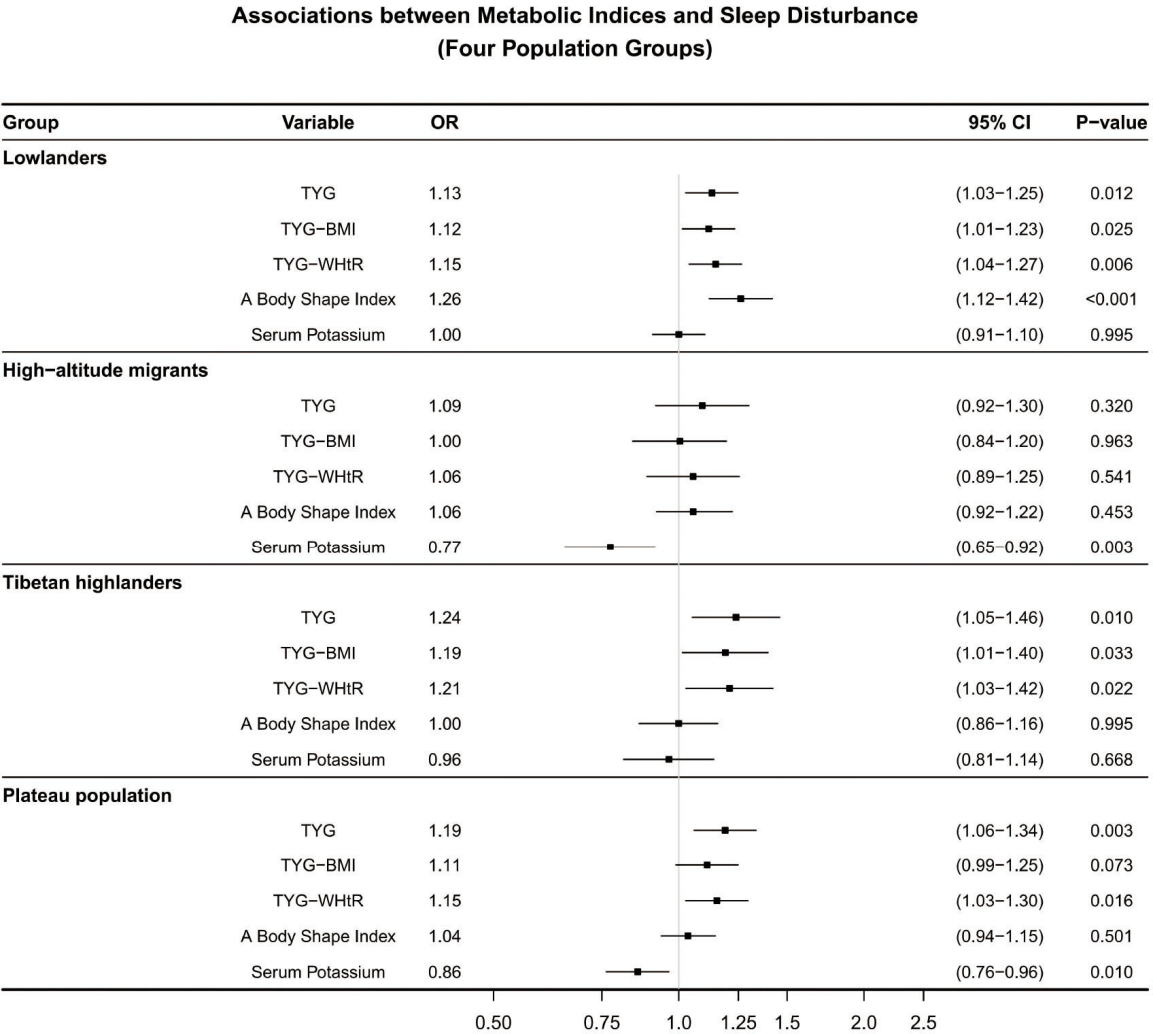
Association between metabolic indices and sleep disturbance across four population groups. Forest plots displaying odds ratios (ORs) and 95% confidence intervals for the associations between metabolic indices (TyG index, TyG-BMI, TyG-WHtR, A Body Shape Index, and serum potassium) and sleep disturbance in lowlanders, high-altitude migrants, and Tibetan highlanders. Models were adjusted for demographic, lifestyle, and psychological covariates.

The restricted cubic spline analyses (**Figure 3**; **Supplementary Figure 3**) revealed that the associations between metabolic indices and sleep disturbance were predominantly linear in most population subgroups, as indicated by nonsignificant P-values for nonlinearity (P > 0.05). Notably, this linear dose-response relationship was consistently observed across various exposure windows. However, a significant nonlinear association was identified for the TyG index specifically within the Tibetan highlanders (P for nonlinearity = 0.021). **Figure 4** provides a comparative summary of the association strengths (ORs) for each metabolic index across the three populations, visually underscoring the divergent results for the high-altitude migrants group. Consistent with the generally modest strength of these associations, the predictive power of all metabolic indices was limited, with area under the receiver operating characteristic curve (AUC) values ranging from 0.571 to 0.628 across populations (**Supplementary Table 1**). Sleep disturbance is too complex (psychological, environmental, and metabolic factors) to be diagnosed by metabolic factors alone, despite their significant statistical associations.

### 3.5 Sensitivity analyses

All sensitivity analyses, including propensity score matching with varying caliper widths (0.15, 0.25, and 0.3) and logistic regression validation, yielded results consistent with the primary analyses, confirming the robustness of our findings (**Supplementary Table 2**).

## 4 Discussion

This comparative study provides systematic confirmation that sleep disorders manifest through distinct risk factors across populations, demonstrating a significant prevalence gradient from lowlanders (14.9%) to combined Plateau population (30.6%, *P* < 0.001), with prevalence of 35.0% in plateau migrants and 27.1% in plateau-native Tibetans (*P* < 0.001). We identified population-specific risk profiles: lowlanders exhibited a broadly metabolic-dominant pattern, with multiple metabolic indices showing significant associations with sleep disorders (TyG index OR = 1.13, 95% CI: 1.03-1.25; TyG-BMI OR = 1.12, 95% CI: 1.01-1.23; TyG-WHtR OR = 1.15, 95% CI: 1.04-1.27; ABSI OR = 1.26, 95% CI: 1.12-1.42). The combined Plateau population showed significant associations with TyG index (OR = 1.19, 95% CI: 1.06-1.33), TyG-WHtR (OR = 1.15, 95% CI: 1.03-1.30), and potassium (OR = 0.86, 95% CI: 0.76-0.96). Plateau migrants demonstrated a psychological-metabolic synergistic pattern, characterized by attenuated metabolic associations but significant potassium association (OR = 0.77, 95% CI: 0.65-0.92), with depression associated with a stronger the TyG-WHtR-sleep disorder association. Highland Tibetans displayed a TyG-related metabolic dominance pattern, maintaining significance only for TyG-related metabolic indicators (TyG index OR = 1.24, 95% CI: 1.05-1.46; TyG-BMI OR = 1.19, 95% CI: 1.01-1.40; TyG-WHtR OR = 1.21, 95% CI: 1.03-1.42) alongside a non-linear TyG threshold effect and potassium-anxiety interaction associated with a weaker anxiety-sleep association. These findings collectively indicate that long-term high-altitude exposure and genetic background (genetic changes caused by long-term altitude exposure) are associated with modified sleep disorder risk profiles.

The prevalence of sleep disorders in the lowland sample of this study (14.9%) is consistent with the pooled estimate (12.4%, 95% CI: 9.0–16.8%) from a recent meta-analysis of 48 studies on general population insomnia based on DSM criteria (50). Similarly, a population-based study in Ningbo, China, using the same PSQI cutoff (>7), reported a prevalence of 8.6% (43); this lower figure may reflect differences in age structure and regional characteristics. In contrast, the prevalence rates were substantially higher in the high-altitude groups of this study (30.6% for the overall plateau population, 35.0% for migrants, and 27.1% for Tibetans). This increase is consistent with a Chinese study that found an overall sleep disorder prevalence of 26.09%, including 41.54% at high altitude (2,300 m) versus 18.76% at low altitude (29). The prevalence gradient—lowest among lowland residents, intermediate among Tibetans, and highest among migrants—aligns with observations that migrants from low-altitude areas typically experience poorer sleep quality than native highlanders (51). Notably, the prevalence rate among high-altitude Tibetans in this study (27.1%) remained far above the lowland average, suggesting that living at high altitudes imposes a residual sleep burden even with long-term genetic adaptation. These comparisons confirm that high-altitude exposure is associated with a significantly increased risk of sleep disorders, and that adaptation patterns (acclimatization vs. genetic adaptation) further modify this risk.

Among the lowlanders, our findings demonstrate consistent positive associations between metabolic indices—including the TyG index, TyG-BMI, TyG-WHtR, and body shape index—and sleep disorder risk, which is consistent with the concept of a metabolically driven phenotype. The TyG index, serving as a validated surrogate marker of insulin resistance (IR), is associated with impaired sleep architecture through multiple pathways. IR disrupts central nervous system homeostasis by altering neurotransmitter balance and circadian regulation, manifesting as prolonged sleep latency and fragmented sleep (10). Concurrently, elevated systemic inflammatory markers may further compromise sleep integrity through HPA axis dysregulation. Composite indices provide enhanced perspectives: TyG-BMI captures dyslipidemia/dysglycemia and overall adiposity, with each unit increase elevating the risk of adverse sleep patterns (14), while TyG-WHtR reflects metabolic dysregulation and central obesity, showing significantly elevated levels across sleep duration patterns in individuals with sleep disorders (52). These observations are consistent with a bidirectional sleep-metabolism relationship wherein sleep abnormalities may exacerbate insulin resistance and promote obesogenic fat distribution, while metabolic dysfunction constitutes a key physiological substrate for sleep disruption. In the lowlanders, metabolic dysregulation—particularly the synergistic interaction between insulin resistance and varied obesity phenotypes—appears to be associated with principal risk factors basis for sleep disturbances.

When analyzing high-altitude populations, we first compared high-altitude residents as a whole with lowland residents. The analysis showed that high-altitude exposure was significantly associated with a higher prevalence of sleep disorders (30.6% vs. 14.9%), consistent with previous reports that sleep disorders are more common in high-altitude regions than in lowland areas (10, 29, 32). The TyG index and TyG-WHtR were also positively associated with sleep disorders in high-altitude populations, aligning with the established link between insulin resistance and sleep disturbances (10). Moreover, serum potassium was associated with lower odds (OR = 0.86) of sleep disturbances in the high-altitude population. In addition, the high-altitude environment as a whole entails both metabolic risks and potential electrolyte-based protective mechanisms. Given that high-altitude populations differ in their adaptation modes—migrants rely primarily on individual acclimatization, whereas Tibetans have evolved genetic adaptations over tens of thousands of years (53) —we further disaggregated the plateau population into migrants and Tibetans for subgroup analyses.

Sleep disorders had the highest prevalence among people who had migrated to high-altitude regions. However, conventional metabolic indicators that were significantly associated with sleep disorders in lowland populations showed weakened or non-significant effects in this group. This pattern does not exclude the role of metabolic factors, but strongly suggests a shift in the primary influencing factors among plateau migrants, wherein other mechanisms may override or mask metabolic influences. The high-altitude environment itself constitutes a potent physiological stressor that directly disrupts sleep architecture. Hypoxia, the defining feature of this environment, activates peripheral chemoreceptors, alters respiratory control, and contributes to sleep-disordered breathing patterns including central and obstructive sleep apnoea (23). With increasing elevation, polysomnographic studies document a redistribution of sleep stages, characterized by increased non-rapid eye movement sleep at the expense of slow-wave sleep (25). Nocturnal monitoring at altitude consistently demonstrates decreased oxygen saturation, reduced N3 sleep, diminished sleep efficiency, and increased sleep fragmentation (54). Beyond these direct environmental effects, our interaction models observed a synergistic pathway specific to highland migrants: a significant positive interaction between depressive symptoms and TyG-WHtR. Depression may disrupt sleep through multiple pathways including HPA-axis dysregulation, neuroendocrine alterations, and pro-inflammatory signaling (55, 56). Concurrently, TyG-WHtR—a composite measure integrating dyslipidemia, dysglycemia, and central adiposity—has been independently linked to sleep disturbances, abnormal sleep duration, and impaired sleep quality in large-scale studies (19, 57, 58). Potential mechanisms include systemic inflammation, HPA-axis dysfunction, and mechanical effects of abdominal obesity on respiratory anatomy (19, 59). Critically, unlike indigenous Tibetan highlanders, migrant populations lack the genetic adaptations that confer resilience to high-altitude stress. This evolutionary mismatch may contribute to attenuated influence of metabolic factors in these groups, in which environmental stressors and psychometabolic interactions-rather than conventional metabolic markers-appear to be dominant determinants of sleep disturbance.

In high-altitude migrants, persistent hypoxic exposure may establish a state of chronic physiological stress, creating conditions where psychological distress (e.g., depressive symptoms) and metabolic-inflammatory pathways (e.g., central obesity) may interact synergistically to disrupt sleep. The pattern observed in these non-adapted highland residents was consistent with a multi-system dysregulation state, wherein distinct risk pathways exhibit abnormal synergistic interactions under sustained environmental challenge. Notably, potassium ions were associated with lower odds of sleep disturbance in this highland migrant population. Under hypoxic stress, adequate potassium availability may help stabilize sleep-wake rhythms by maintaining neuronal membrane potential and attenuating sympathetic nervous system hyperexcitability (60). This stabilizing mechanism assumes particular importance in high-altitude environments, where hypoxia inherently disrupts autonomic balance (61).

In contrast to migrants, the genetically adapted Tibetan highlanders exhibited a distinct pattern. Having lived on the Tibetan Plateau for over thirty millennia, Tibetan populations possess distinct physiological and genetic adaptations to chronic hypoxia (62). Extending this evolutionary framework to sleep research, we found that among Tibetan highlanders, only TyG-related metabolic indicators (TyG index, TyG-BMI and TyG-WHtR) maintained significant associations with sleep disorders. Specifically, the TyG index showed a unique J-shaped dose-response relationship with sleep disorder risk (P for nonlinearity < 0.05). Below the threshold of approximately 0.85, sleep disorder risk remained stable, while beyond this point, risk escalated sharply: a pattern absent in both lowland and non-Tibetan high-altitude populations. This suggests that through prolonged natural selection, Tibetans may have established a refined homeostatic set-point: effective compensatory mechanisms are associated with maintenance of sleep homeostasis below the threshold, while exceeding it is associated with rapid decompensation and disproportionately increased risk. The genetic architecture underlying this adaptation is well-characterized. Tibetan populations show distinctive signatures of positive selection in hypoxia-inducible factor pathway genes (including *EPAS1* and *EGLN1*) *(*33). EPAS1 shows altitude-associated allele frequencies in Tibetans, and a 3.4 kb deletion is linked to low hemoglobin (62); protective Denisovan-introgressed haplotypes are enriched at altitude (63). EGLN1 enhances oxygen affinity (38, 64), and PPARA may compensate HIF signaling (65). These adaptations optimize oxygen delivery, enabling hypoxic homeostasis (66). We hypothesize this system underlies the TyG-sleep threshold we observed, providing an evolutionary perspective. As we did not measure these markers, our interpretations are hypothesis-generating and require genotyping.

To elucidate the complex interactions between psychological and physiological dimensions of sleep disorders, we conducted detailed interaction analyses. Our results showed a significant interaction between serum potassium and anxiety symptoms specifically in the Tibetan population (interaction OR = 0.59, 95% CI: 0.36–0.97, P = 0.040), which was associated with a weaker effect of anxiety on sleep disturbance. No comparable moderating effects reached statistical significance in other populations. These results extend previous work documenting the sleep-impairing role of anxiety. Both persistent daytime anxiety and acute pre-sleep worry reliably predict impaired sleep efficiency, reduced total sleep time, and difficulties with sleep initiation (61). Electroencephalographic evidence further links anxiety to reduced deep sleep (N3) duration (67). Mechanistically, low potassium availability may lower the threshold for anxiety-related neural excitability by impairing inhibitory neuronal function (68), while epidemiological studies consistently identify inadequate dietary potassium as an independent risk factor for anxiety (69, 70). Our findings suggest an significant evolutionary perspective to this pathway: the Tibetan highlanders’s long-term adaptation to high altitude may have contributed to potassium ion balance serving as a physiological buffering mechanism against psychological stress. The consistent observation of potassium-related associations—both as a factor associated with lower sleep disturbance in highland migrants and as a factor associated with a weaker anxiety-sleep link in adapted Tibetans—points to electrolyte homeostasis as a promising, modifiable target for sleep health preservation in high-altitude populations.

These findings provide epidemiological foundation for precision sleep medicine, supports a shift from one-size-fits-all approaches to population-specific interventions: lowlanders could benefit from metabolic management with TyG monitoring; highland migrants may require integrated management of depression-central obesity comorbidity; and Tibetan highlanders could consider TyG index monitoring against the 0.85 threshold while exploring potassium homeostasis as a factor associated with lower risk. Future studies should prioritize genetic dissection of these relationships by directly testing associations between established high-altitude adaptation genes (e.g., EPAS1, EGLN1, PPARA) and the identified metabolic-psychological phenotypes, particularly the TyG index threshold effect and potassium-anxiety interaction.

## Limitations

This study has several limitations that warrant consideration. The cross-sectional design prevents causal inferences about the observed relationships, and the reliance on subjective sleep measures rather than objective polysomnography may introduce reporting bias, despite using validated scales. Although we comprehensively adjusted for known confounders through propensity score matching and multivariate models, residual confounding from unmeasured genetic, environmental, or behavioral factors cannot be excluded. Despite these limitations, this work provides robust hypothesis-generating evidence by identifying population-specific patterns and interactions that establish a clear framework for future research. Subsequent longitudinal studies, intervention trials, and mechanistic investigations incorporating objective sleep measurements, and genetic data can now strategically test and refine the proposed causal pathways.

## Conclusion

Our findings show a clear spectrum of risk for sleep disturbance across populations, which varies with environmental and adaptive backgrounds. General lowlanders showed broad susceptibility associated with multiple metabolic factors. For high-altitude migrants, these metabolic links were not evident, and this pattern may reflect stronger environmental and psychological influences. Particularly notable was the profile of the genetically adapted Tibetan highlanders, who exhibited a focused “TyG-related metabolic” phenotype, with sleep disturbance associated specifically with key insulin resistance indicators. This distinct gradient offers a powerful framework for considering how human populations may exhibit divergent disease manifestations under environmental pressure.

## Data Availability

The original contributions presented in the study are included in the article/**Supplementary Material**, further inquiries can be directed to the corresponding author.
