# Supplementary material for: Environmental adaptation and sleep disturbance: a cross-sectional study reveals distinct metabolic risk profiles in long-term high-altitude residents versus the general population
Source: Front Psychiatry. 2026 Jul 3;17:1789618. doi: 10.3389/fpsyt.2026.1789618 (PMC13376246; doi:10.3389/fpsyt.2026.1789618)
Supplement: Supplementary file 1 [file DataSheet1.pdf]

Supplementary Appendix

Page 1 ... Table of Contents
Page 2 ... Supplementary Figure S1. Directed acyclic graph (DAG) of the causal framework.
Page 3 ... Supplementary Figure S2. Love plot demonstrating standardized mean differences (SMDs) before and after propensity score matching.
Page 4 ... Supplementary Figure S3. Restricted cubic spline plots depict the dose-response relationships between metabolic indices and sleep disturbance risk across three population groups
Page 5 ... Supplementary Table S1. Predictive Performance of Various Exposure Variables for Sleep Disorder: Results from Receiver Operating Characteristic (ROC) Curve Analysis
Page 6 ... Supplementary Table S2. Sensitivity Analysis: Association of Various Exposure Variables with Sleep Disorder Across Different Propensity Score Matching Calipers
Page 7 ... Supplementary Document 1. Statistical Review Declaration

Supplementary Figure S1. Directed acyclic graph (DAG) of the causal framework.

X: Metabolic indices (exposure). Y: Sleep disturbance (outcome). M: Psychological symptoms (depression and anxiety). C: Confounders (age, sex, marital status, education, smoking, alcohol, BMI, LOT, SWLS, loneliness, hypertension). Solid arrows: Causal paths. Confounders (C) influence both X and Y. X affects Y directly (X → Y) and through M (X → M → Y). Red dashed bidirectional arrow: Effect modification (interaction). Interaction terms (X × M) were included to test whether depression or anxiety moderates the association between metabolic indices and sleep disturbance, after adjustment for all confounders.

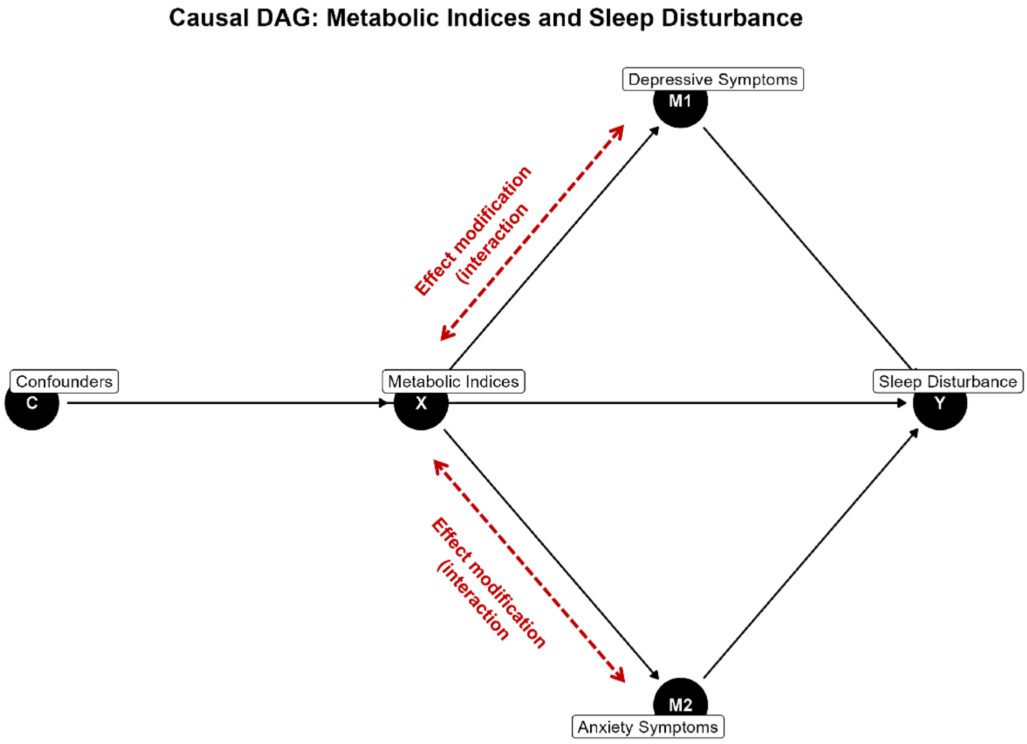

Supplementary Figure S2. Love plot demonstrating standardized mean differences (SMDs) before and after propensity score matching.

Purple dashed line indicates the SMD threshold of 0.1 for adequate balance. Circles represent SMDs before matching; triangles represent SMDs after matching. All matching variables (age, sex, education, marital status, smoking, drinking, BMI, hypertension) achieved SMD < 0.1 after matching, indicating good balance between lowlanders and the combined high-altitude population.

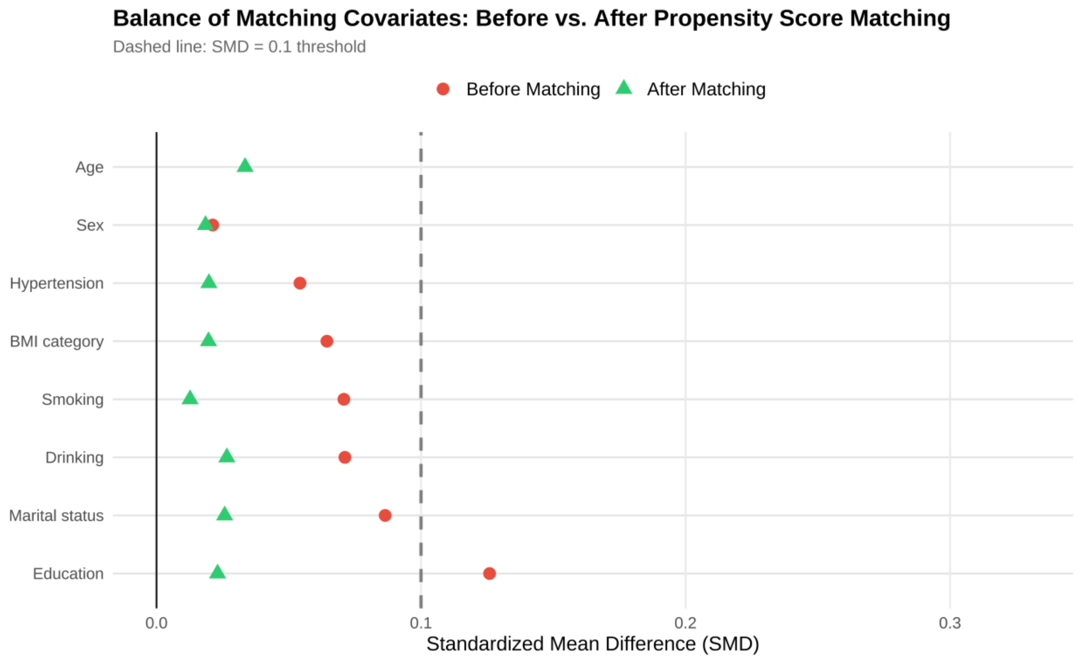

Supplementary Figure S3. Restricted cubic spline plots depict the dose-response relationships between metabolic indices and sleep disturbance risk across three population groups

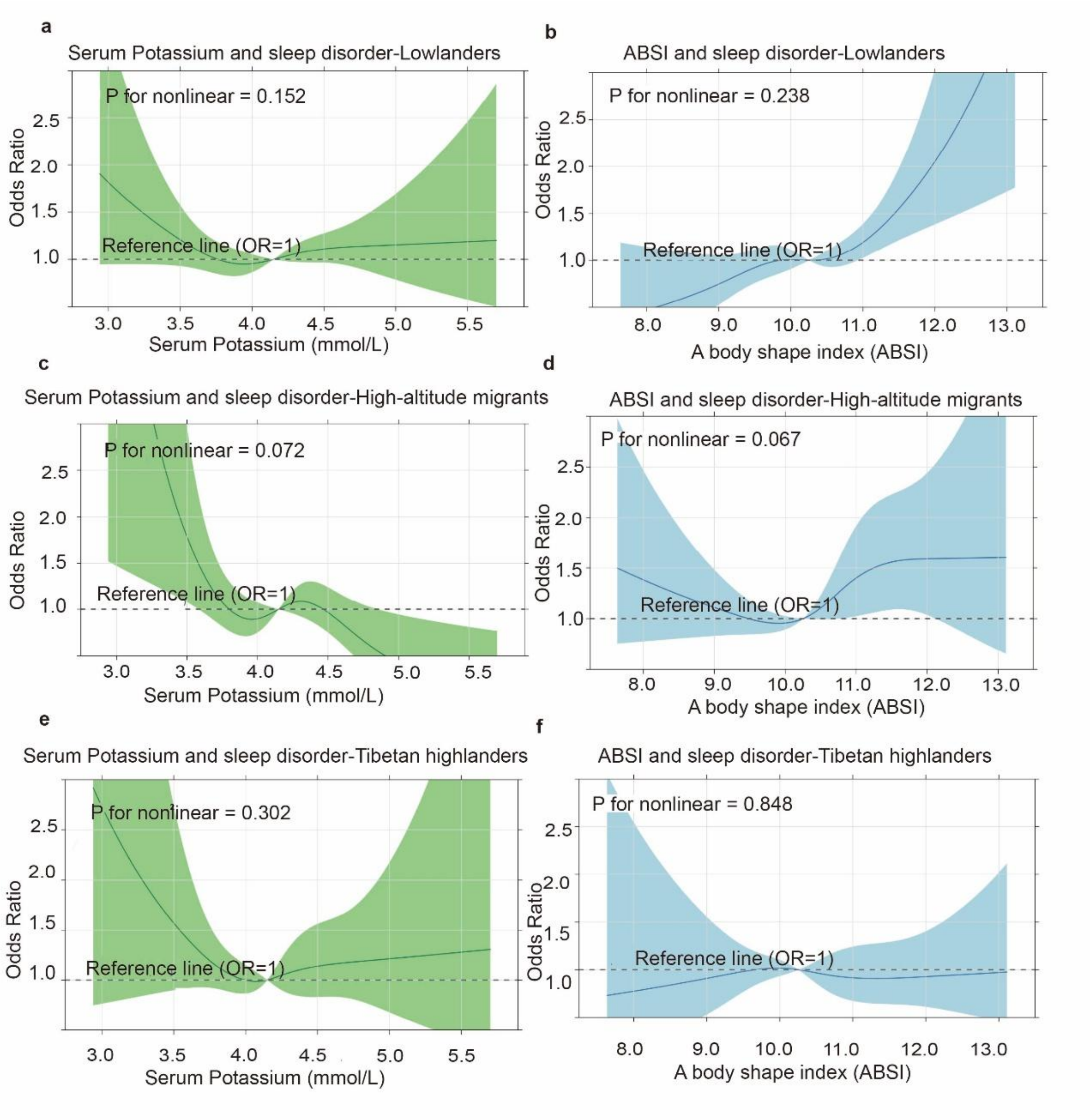

（a）Serum Potassium and sleep disturbance risk in lowlanders; (b) ABSI and sleep disturbance risk in lowlanders; (c) Serum Potassium and sleep disturbance risk in lowlanders; (d) ABSI and sleep disturbance risk in high-altitude migrants; (e) Serum Potassium and sleep disturbance risk in high-altitude migrants; (f) ABSI and sleep disturbance risk in high-altitude migrants. All models are adjusted for covariates (e.g., age, sex, psychological scores). The solid line represents the estimated odds ratio, and the shaded area indicates the 95% confidence interval.

Supplementary Table S1. Predictive Performance of Various Exposure Variables for Sleep Disorder: Results from Receiver Operating Characteristic (ROC) Curve Analysis

Model	Population	AUC	AUC_95 CI	Thresh old	Sensitiv ity	Specific ity	Case_Nu mber	Control_Nu mber
TYG	Lowlanders	0.612	0.586 - 0.638	0.14	0.695	0.477	514	2941
TYG	High-altitude migrants	0.613	0.571 - 0.655	0.338	0.706	0.487	269	489
TYG	Tibetan highlanders	0.586	0.545 - 0.626	0.228	0.811	0.323	270	700
TyG-BMI index	Lowlanders	0.611	0.585 - 0.637	0.142	0.681	0.491	514	2941
TyG-BMI index	High-altitude migrants	0.61	0.568 - 0.652	0.329	0.766	0.429	269	489
TyG-BMI index	Tibetan highlanders	0.579	0.539 - 0.62	0.315	0.407	0.721	270	700
TyG-WHtR	Lowlanders	0.613	0.586 - 0.639	0.138	0.698	0.466	514	2941
TyG-WHtR	High-altitude migrants	0.611	0.569 - 0.653	0.323	0.77	0.415	269	489
TyG-WHtR	Tibetan highlanders	0.582	0.542 - 0.623	0.32	0.393	0.744	270	700
A Body Shape Index	Lowlanders	0.617	0.59 - 0.643	0.139	0.673	0.496	514	2941
A Body Shape Index	High-altitude migrants	0.611	0.569 - 0.652	0.345	0.669	0.509	269	489
A Body Shape Index	Tibetan highlanders	0.574	0.533 - 0.615	0.287	0.559	0.577	270	700
Serum potassium	Lowlanders	0.609	0.583 - 0.635	0.157	0.584	0.591	514	2941
Serum potassium	High-altitude migrants	0.628	0.587 - 0.669	0.308	0.807	0.399	269	489
Serum potassium	Tibetan highlanders	0.571	0.53 - 0.613	0.305	0.481	0.659	270	700

Note: This table presents the predictive performance of various exposure variables for sleep disorder, stratified by population subgroup. The performance was evaluated using Receiver Operating Characteristic (ROC) curve analysis, with models adjusted for Age, Sex, Marital status, Education level, Smoking status, Alcohol consumption, scores on the Life Orientation Test (LOT), the Satisfaction with Life Scale (SWLS), the Loneliness Scale (LONLY), hypertension, anxiety symptoms (assessed by the GAD-7), and depressive symptoms (assessed by the PHQ-9). The Area Under the Curve (AUC) represents the overall predictive accuracy. The optimal threshold was selected to maximize the sum of Sensitivity and Specificity. Case_Number and Control_Number refer to the sample sizes of the sleep disorder and non-sleep disorder groups used in the analysis, respectively. The generally low AUC values across all groups indicate limited utility of these indices as standalone diagnostic tools, consistent with the multifactorial nature of sleep disturbance.

Supplementary Table S2. Sensitivity Analysis: Association of Various Exposure Variables with Sleep Disorder Across Different Propensity Score Matching Calipers

		Lowlanders		High-altitude migrants		Tibetan highlanders	
Caliper (SD)	Independent variable	OR (95%CI)	P.value	OR (95%CI)	P.value	OR (95CI)	P.value
0.15	TyG index	1.106(1.003,1.219)	0.042	1.079(0.905,1.285)	0.395	1.233(1.047,1.450)	0.012
0.15	TyG-BMI index	1.010(0.906,1.078)	0.780	0.985(0.670,1.440)	0.937	1.400(0.993,1.964)	0.053
0.15	TyG-WHtR	1.126(1.020,1.242)	0.018	1.040(0.873,1.237)	0.660	1.200(1.020,1.410)	0.027
0.15	A Body Shape Index	1.175(1.052,1.326)	0.006	1.058(0.903,1.253)	0.497	0.987(0.834,1.166)	0.879
0.15	Serum potassium (K^+)	1.075(0.974,1.186)	0.148	0.804(0.676,0.953)	0.013	0.996(0.838,1.182)	0.962
0.25	TyG index	1.105(1.001,1.217)	0.045	1.067(0.895,1.270)	0.468	1.258(1.070,1.478)	0.005
0.25	TyG-BMI index	1.009(0.904,1.078)	0.789	0.958(0.654,1.398)	0.826	1.495(1.061,2.102)	0.021
0.25	TyG-WHtR	1.125(1.018,1.241)	0.020	1.030(0.865,1.226)	0.735	1.218(1.037,1.429)	0.016
0.25	A Body Shape Index	1.176(1.053,1.328)	0.006	1.065(0.909,1.262)	0.446	0.971(0.818,1.15)	0.735
0.25	Serum potassium (K^+)	1.076(0.975,1.186)	0.146	0.800(0.673,0.947)	0.011	1.004(0.845,1.192)	0.963
0.3	TyG index	1.105(1.001,1.217)	0.045	1.086(0.914,1.289)	0.350	1.260(1.072,1.481)	0.005
0.3	TyG-BMI index	1.009(0.904,1.078)	0.789	0.992(0.679,1.443)	0.968	1.482(1.051,2.083)	0.024
0.3	TyG-WHtR	1.125(1.018,1.241)	0.020	1.052(0.886,1.247)	0.564	1.214(1.034,1.425)	0.018
0.3	A Body Shape Index	1.177(1.053,1.329)	0.006	1.073(0.916,1.271)	0.398	0.964(0.812,1.141)	0.672
0.3	Serum potassium (K^+)	1.076(0.975,1.186)	0.146	0.809(0.681,0.958)	0.015	1.004(0.845,1.192)	0.963

Note: This table presents the results of sensitivity analyses evaluating the robustness of the associations between metabolic indices and sleep disturbance. Analyses were conducted using logistic regression on samples generated by propensity score matching with varying caliper widths (0.15, 0.25, and 0.30 of the propensity score standard deviation). All models were adjusted for age, sex, marital status, education level, smoking status, alcohol consumption, scores on the Life Orientation Test (LOT), the Satisfaction with Life Scale (SWLS), the Loneliness Scale (LONLY), hypertension, anxiety symptoms (GAD-7), and depressive symptoms (PHQ-9). Analyses were conducted separately in three subgroups: Lowlanders, High-altitude migrants, and Tibetan highlanders. Abbreviations: OR, Odds Ratio; CI, Confidence Interval

Supplementary Document 1. Statistical Review Declaration

This document is a signed declaration by Prof. Deying Kang (Center of Biostatistics, Design, Measurement and Evaluation, West China Hospital, Sichuan University), confirming the appropriateness of the statistical methods used in this study, including propensity score matching, multivariable logistic regression, restricted cubic spline analysis, interaction testing, and multiple comparison corrections.

Certificate of Biostatistics

18 May 2026

To whom it may concern

Title of Study: Environmental adaptation and sleep disturbance: A cross-sectional study reveals distinct metabolic risk profiles in long-term high-altitude residents versus the general population

Chief investigator:

This letter is to confirm that I have read the ethics application prepared for this study, and that in my opinion the statistical methods and techniques mentioned are appropriate for the research.

Please contact me if you have any queries.

Sincerely,

I have reviewed the statistical methods and results of this study as presented in the revised manuscript. In my opinion, the statistical approaches used are appropriate for the research objectives and the cross-sectional study design. The key procedures include propensity score matching to balance baseline covariates, multivariable logistic regression to assess associations between metabolic indices and sleep disturbance, restricted cubic spline analysis to examine dose- response relationships, interaction analysis to evaluate effect modification, and sensitivity analyses to test the robustness of the findings.

Special note: This certificate is solely used to scientifically and reasonably prove the methods of statistical analysis and does not serve as proof of the authenticity and accuracy of the original data.

Deying Kang, Prof. , Center of Biostatistics, Design, Measurement and Evaluation, West China Hospital of Sichuan University, Chengdu 610041, China.

E-mail:deyingkang@126.com
Telephone:+86 028 8542 2851
